# Primary intra-osseous Hybrid Schwannoma-Perineurioma in the mandible

**DOI:** 10.4317/jced.57035

**Published:** 2020-09-01

**Authors:** Mariana-Lobo Bergamini, Luanna-Priscilla-Montenegro Noberto, Gabriel-Barroso-Marocco-de Abreu Torres, Haroldo-Arid Soares, Fabiana Martins, Andre-Luiz-Ferreira Costa, Suzana-Orsini-Cantanhede-Machado de Souza, Paulo-Henrique Braz-Silva

**Affiliations:** 1 Department of Stomatology, School of Dentistry, University of São Paulo, São Paulo, Brazil; 2 Division of Odontology, Municipal Hospital Carmino Caricchio, São Paulo, Brazil; 3 Department of Dentistry, University of Santo Amaro, São Paulo, Brazil; 4 Postgraduate Program in Dentistry, Cruzeiro do Sul University, São Paulo, Brazil; 5 Laboratory of Virology, Institute of Tropical Medicine of São Paulo, School of Medicine, University of São Paulo, São Paulo, Brazil

## Abstract

Benign nerve sheath tumours include perineuriomas, schwannomas and neurofibromas. Hybrid schwannoma-perineurioma represents a cutaneous, subcutaneous or occasionally intra-osseous tumour with schwannian cytomorphology and perineurioma-like architecture consisting of a mixture of both types of cells. These tumours can develop at any age and there is no gender-predilection. Tongue is the most frequently affected site, followed by palate, mouth floor, jugal mucosa, lips and, more rarely, mandible. We present a case of hybrid tumour with schwannoma-perineurioma morphology located on the right mandibular body (intra-osseous) of a 54-year-old female patient. The tumour was symptomatic and evolving for six months. Microscopically, it was encapsulated and highly cellularised, presenting fascicular aspect and exhibiting scant mitotic activity. The tumour consisted of distinct cellular populations involving fusiform cells, cells with wavy and hyperchromatic nucleus or even epithelioid cells. Positive immunostaining for S-100 and epithelial membrane antigen (EMA) was observed. The lesion was completely removed under general anaesthesia, with the patient showing no clinical or radiographic sign of relapse after two-year follow-up. Despite the limited knowledge on the pathogenesis of Hybrid Schwannoma-Perineurioma, these tumours seem to present a non-aggressive biological behaviour. Conservative surgery provides adequate solution without recurrence, even after a long-term follow-up.

** Key words:**Nerve sheath tumours, Schwannoma, Perineurioma, Immunohistochemistry.

## Introduction

Hybrid tumours of peripheral nerve sheath are benign mixed neoplasias with characteristics of more than one type of nerve sheath, encompassing a spectrum of well-defined clinical-pathological entities, such as schwannomas, neurofibromas and perineuriomas (1-3). In general, these three tumours can be differentiated depending on their clinical variations, cell composition, growth pattern and immunophenotypic profile. Hybrid tumours of peripheral nerve sheath have been increasingly reported elsewhere, involving more than one histological type, such as schwannoma-neurofibroma, neurofibroma-perineurioma or schwanooma-perineurioma (2-4).

Schwannomas, also known as neurilemomas or neurinomas, are a group of peripheral nerve sheath tumors consisting of Schwann neoplasic cells with variable morphological characteristics (5). Approximately 5% of the schwannomas appear in the head and neck region, but only 1% is localised in the oral cavity where tongue is the most frequently affected site, followed by palate, mouth floor, jugal mucosa, lips, gingiva and, more rarely, mandible. Although this tumour may appear at any age, it is more commonly seen in the fourth decade of life (6,7).

In general, schwannomas are asymptomatic encapsulated tumours with slow growth which usually appear in association with a nerve trunk and, as they grow in size, they push the nerve aside. Intra-osseous tumours can cause bone expansion, pain and paresthesia. These lesions are more frequently associated with sensory nerves (8,9).

Perineuriomas are benign neoplasias with advanced perineurial differentiation, being first described in 1978. These tumours can be classified into two subtypes, namely, intra-neural and extra-neural depending on their clinical and morphological characteristics (10,11). These tumours occur more commonly in the subcutaneous tissues of the trunk and limbs, but can also appear in the dermis and deep soft tissues. Oral perineuriomas have been little reported in the literature, and the clinical-pathological profiles of these neoplasias in the oral cavity have not been well established yet (12,14-17).

Hybrid schwannoma-perineurioma tumours are circumscribed, but they are usually non-encapsulated and present lamellar or storiform architecture similar to a perineurioma. However, they have predominantly a cytomorphology of schwannoma consisting of fusiform cells with wavy and thin nuclei, pale eosinophilic cytoplasm and indistinct cell boundaries (18,19).

The objective of the present study was to present a case of primary intra-osseous tumour with hybrid schwannoma-perineurioma morphology.

## Case Report

**Figure 1:**
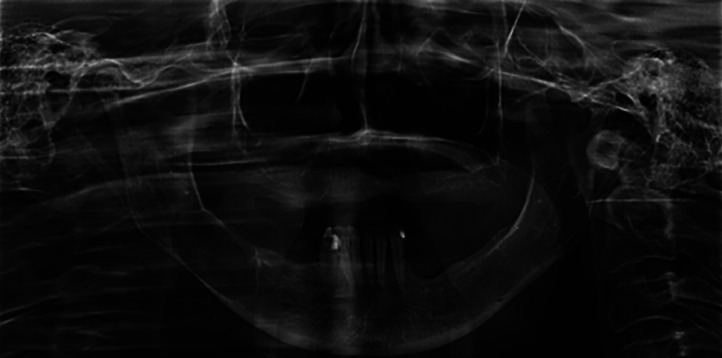
Panoramic radiography showing radiolucent expansile lesion measuring 1.5 cm in diameter located in the right mandibular body.

A woman attended the Carmino Caricchio Municipal Hospital complaining of diffuse pain in the region of the right mandibular body and of numbness in the right lower lip for about six months. The patient reported that she had undergone extraction of a molar tooth and after the procedure there was a significant worsening of the symptoms. Radiographic examination showed presence of a radiolucent lesion measuring 1.5 cm in diameter located in the right mandibular body (Fig. 1). The patient was then submitted to excisional biopsy and the diagnostic hypothesis was a tumour of mesenchymal origin.

**Figure 2:**
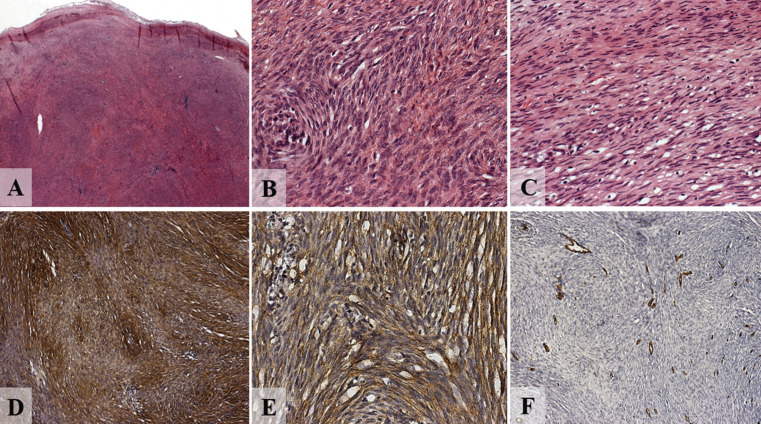
A) Low power view showing fragment of tumor of neural origin, encapsulated, highly cellularized and of fascicular aspect (hematoxylin and eosin [H&E], magnification X25); B) this compound being either by fusiform cells, sometimes by cells that exhibit a wavy and hyperchromatic nucleus; C) or even epithelioid cells, with little mitotic activity being noted (H&E, magnification X400).; D) immunohistochemistry of hybrid neural tumor. The schwannian areas are diffusely positive for S100 (magnification X100); e, epithelial membrane antigen (EMA) is diffusely positive in the spindle cells (magnification X400); f, negative immunostaining for CD34 (magnification X100).

The biopsy material was sent to Oral and Maxillofacial Surgical Pathology Service, Department of Stomatology, School of Dentistry, University of Sao Paulo. Microscopic analysis revealed fragment of neural tumour, encapsulated, highly cellularised and with fascicular aspect, consisting of fusiform cells with both wavy and hyperchromatic nuclei, or even of epithelioid cells, with scant mitotic activity. Immunohistochemical examination revealed a strong positive labelling for anti S-100 anti-body (Flex Polyclonal Rabbit, anti-S100, ready-to-use [Link], DAKO Corporation, Carpinteria, USA) and epithelial membrane antigen (Monoclonal Mouse, anti-human Epithelial Membrane Antigen, clone E29, DAKO Corporation, Carpinteria, USA). CD34 negative immunostaining was observed (Monoclonal Mouse anti-human clone QBEnd-10, Dako Corporation, Carpinteria, USA) (Fig. 2).

**Figure 3:**
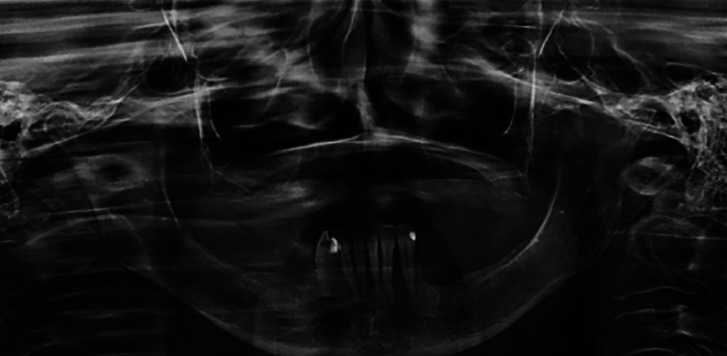
Panoramic radiography after two years of follow-up showing absence of the lesion.

After the diagnosis of primary intra-osseous Hybrid Schwannoma-Perineurioma, the patient underwent treatment in the hospital. A panoramic radiography was performed after two years, showing no evidence of relapse of the lesion (Fig. 3).

## Discussion

The majority of the benign peripheral nerve sheath tumours can be classified into a given category depending on their different morphological and immunohistochemical characteristics. In 1998, Feany *et al.* (2) reported for the first time nine cases of neurofibroma with Schwann cell differentiation. Since then, cases with hybrid characteristics of more than one type of benign peripheral nerve sheath tumour have been successively reported and denominated as hybrid peripheral nerve sheath tumours (HPNST) (19).

Benign peripheral nerve sheath tumours consist of a heterogeneous group of lesions sharing nerve sheath differentiation, but which are clinically, morphologically and immunohistochemically distinct (1). Among them, schwannoma and neurofibroma are the most common histological types, whereas perineurioma is considerably more seldom (20).

These peripheral nerve sheath tumours, in general, are composed of only one histological type. Nevertheless, although tumours with hybrid characteristics of more than one histological type are rare, they have been increasingly more reported in the past years (13,21). Two forms are represented on the basis of architectonic structures, namely: biphasic tumours (known as neurofibroma-schwannoma or schwannoma-perineurioma), in which two distinct areas are clearly indentified (2-3), and monophasic tumours (known as hybrid schwannoma-perineurioma) (12,13), in which two distinct components are closely intermingled (2-4).

Schwannomas are encapsulated tumors consisting of Schwann cells with a biphasic pattern of dense and loose cellular areas with fusiform nuclei (22,24). Perineuriomas, the rarest lesion in the major triad of peripheral nerve sheath tumours (PNST), are similarly composed of one type of cell only: neoplastic perineurial cell (12,14-18). Clinically, intraneural perineuriomas have a presentation similar to that of schwannomas, but they can be immunohistochemically distinguished (22-24).

Perineuriomas were positive for EMA and claudin-1, but negative for S100 (22,23). Extra-neural perineuriomas present as extensive soft-tissue masses which can be confirmed by the presence of perineural cell differentiation and perineurial markers claudin-1 and GLUT-1 (12,14-18).

## Conclusions

Benign nerve sheath tumours with predominantly a cytomorphology of schwannoma and perineurioma-type architecture comprise a mixture of both types of cells. Histological aspects can suggest a diagnosis, but the use of immunomarkers is needed for confirmation. Despite the limited knowledge on their pathogenesis, these tumours seem to present a non-aggressive biological behaviour. Conservative surgery provides adequate solution without recurrence, even after a long-term follow-up.
